# Primary lung lymphoma involving the superior vena cava

**DOI:** 10.1186/1477-7819-10-131

**Published:** 2012-06-30

**Authors:** Sen Wei, Xin Li, Xiaomin Qiu, Honglin Zhao, Gang Chen, Jun Chen, Qinghua Zhou

**Affiliations:** 1 Department of Lung Cancer Surgery, Tianjin Key Laboratory of Lung Cancer Metastasis and Tumor Microenvironment, Tianjin Medical University General Hospital, Tianjin, 300052, China

**Keywords:** Primary lung lymphoma, Lobectomy, Superior vena cava

## Abstract

Primary lung lymphoma (PLL) presenting as a primary pulmonary lesion is rare and usually affects elderly people. Here we describe a 25-year-old Chinese man diagnosed with primary lung lymphoma, which presented as a huge lung tumor mimicking a primary lung cancer and involving the superior vena cava. He underwent double-sleeve reconstructions of bronchus and pulmonary arteries with right upper- and middle-lobe lobectomy along with replacement of the superior vena cava with a graft, and was then given standard chemotherapy of CHOP plus Rituximab. The patient has been well, showing no local recurrence or distal metastasis during a 27-month follow-up.

## Background

Primary lung lymphoma (PLL) is a rare distinct entity (0.4% of all lymphomas) that usually affects elderly patients [1-3]. Generally, these cases are difficult to diagnose accurately because of a nonspecific clinical and radiological presentation. On the other hand, they have relatively satisfactory outcomes, especially in cases amenable to surgical resection. Owing to the rarity of PLL, standard treatment protocols have not yet been optimized, and there is no guideline as to when surgery is indicated.

Herein, we present a young patient with a rare primary pulmonary lymphoma (diffuse large B cell lymphoma, DLBCL) mimicking a primary lung cancer and involving the superior vena cava. He was treated by double-sleeve reconstructions of bronchus and pulmonary arteries with right upper and middle lobe lobectomy, and replacement of the superior vena cava with a graft.

## Case presentation

A 25-year-old Chinese male was admitted because of respiratory symptoms and a large mass on the computed tomography (CT) scan suggestive of primary lung carcinoma. This patient presented with 45 days of an irritating dry cough and right chest pain without other abnormal findings such as fever, wheezing, hemoptysis, or superior vena cava syndrome. He had no smoking history and no family history of lung cancer. A review of systems was noncontributory. On admission, peripheral blood count, serum chemistry, and urinalysis were normal.

**Figure 1:**
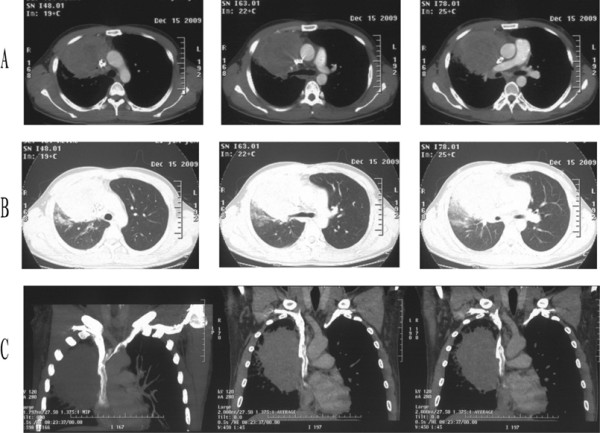
**Enhanced chest CT scan before operation.** (**A**) In the mediastinal window, the CT scan revealed the tumor encroaching on the superior vena cava (right panel), surrounding the right upper lobe bronchus (middle panel), and invading the right pulmonary artery (left panel). (**B**) In the lung window. (**C**) In the mediastinal window, coronal.

An enhanced chest CT scan revealed a huge pulmonary tumor over the right upper lung field with mediastinal and hilar lymphadenopathy surrounding the right upper lobe bronchus. Angiography showed encroachment on the superior vena cava (Figure 1). Abdominal CT, MRI scan of the brain, and a bone scan were all normal. Bronchoscopy showed a subsegmental bronchus of the right upper lobe being pressured from outside without mucosal invasion, and bronchoscopic biopsy did not show any malignancy. Before hospitalization, the patient had undergone a CT-guided fine-needle aspiration biopsy in another medical center and a preoperative diagnosis of squamous cell carcinoma was made. This conclusion later proved to be a misdiagnosis.

**Figure 2:**
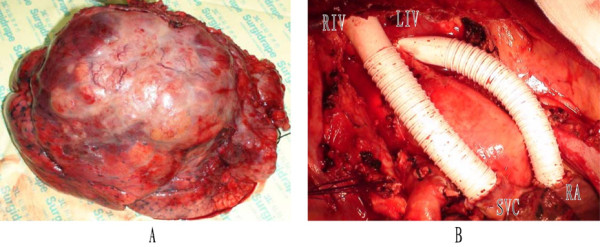
**The images during the operation.** (**A**) Gross features of the 15-cm mass in right upper-middle lobes. (**B**) SVC grafts: the graft on the left is from the right innominate vein (RIV) to the superior vena cava (SVC); the graft on the right is from the left innominate vein (LIV) to the right atrium (RA).

On 21 December, 2009, after establishment of a venous bypass between the right internal jugular vein and the right femoral vein, a surgical exploration was performed through a standard posterolateral incision under general anesthesia. There was a huge tumor (18 × 16 × 15 cm^3^) located in the right upper lobe surrounding the roots of the right upper lobe bronchus and invading the surrounding tissues and organs, including part of the right middle lobe, the middle and lower portion of the superior vena cava, the right side of the pericardium, the right phrenic nerve, and the trunk of right pulmonary artery (Figure 2A). Several oval nodules found at the same time in the middle lobe were suspected to be metastatic lesions. Mediastinal and hilar lymph nodes were enlarged and had a tendency to integration. Intraoperative frozen section of lymph node sampling proved all of them to be metastatic poorly differentiated carcinoma.

**Figure 3:**
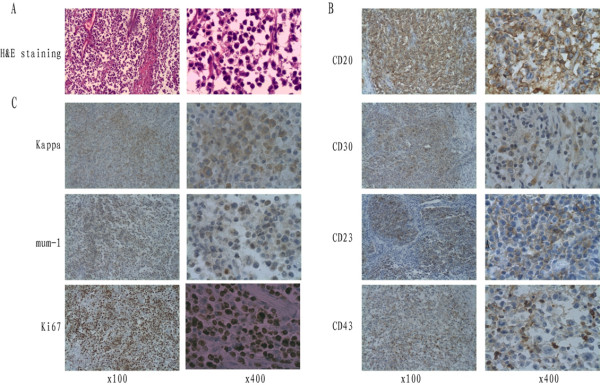
**Histopathological images (×100 and ×400).** (**A**) Hematoxylin and eosin (H&E) staining of primary lung lymphoma. (**B**) Immunohistochemical staining of primary tumor with antibodies to CD20, CD30, CD23, and CD43. (**C**) Immunohistochemical staining of primary tumor with antibodies to Kappa, mum-1, and Ki67.

Based on these findings, sleeve lobectomy of the right upper and middle lobes was performed successfully to achieve complete tumor resection. Portions of the surgical procedure were sophisticated, including sleeve resection and reconstruction of the right bronchus and pulmonary artery, reconstruction of the superior vena cava, partial resection of the pericardium, and systematic mediastinal lymphadenectomy (Figure 2B). Postoperative histopathological assessment of the huge lobulated gray-yellow lung mass revealed that the tumor was situated in the lung parenchyma and involved visceral pleura. The tumor cells exhibited large vesicular nuclei and frequent mitoses without tumor necrosis. Immunohistochemically, the tumor cells expressed CD20, CD23, CD30, CD43, Kappa, and MUM1, but not CD117, TdT, CD10, or Bcl-6. The proliferation fraction as determined by staining with Ki-67 was 80%. Based on these findings, the diagnosis of diffuse large B cell lymphoma (Figure 3) was made. The diagnosis of PPL was based on characteristic histological and immunophenotypical features according to the Kiel classification and the WHO classification [4]. Mediastinal [nos. #2, 3, 4, 7 and 9] and hilar [nos. #10] lymph nodes contained tumor. All of the negative surgical margins (bronchus, pulmonary artery, and superior vena cava) were confirmed. The tumor was determined to be stage IIE by the Ann Arbor classification system [5].

**Figure 4:**
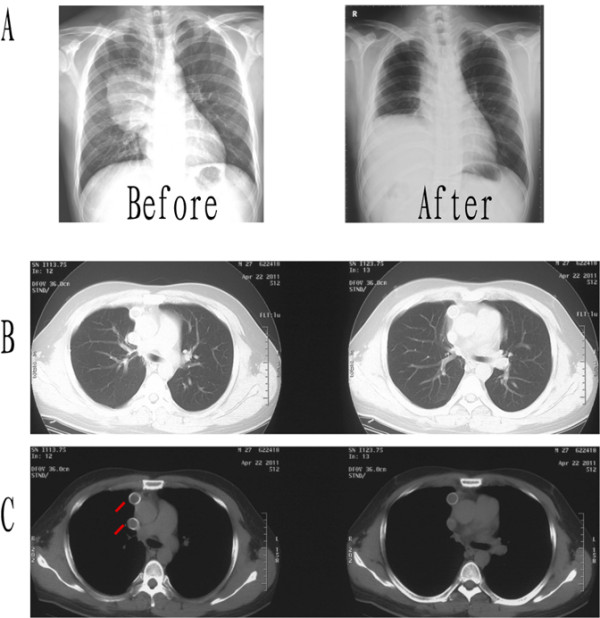
**Chest X-ray and CT scan after operation.** (**A**) The chest x-ray images of the patient before/after operation. (**B** and **C**) The chest CT scan 16 months after the operation. The red lines show the SVC replacement graft.

Five weeks after the operation, the patient received adjuvant therapy with CHOP (cyclophosphamide, Adriamycin, vincristine and prednisone) and Rituximab (a humanized monoclonal anti CD-20 antibody) for six cycles. For economic reasons, he did not receive maintenance therapy of Rituximab. After a 27-month follow-up period, as shown in Figure 4 for16-month after operation, the patient is well and without evidence of locally recurrent or distal disease.

## Discussion

Extranodal lymphomas account for 24% to 48% of NHL, and primary pulmonary lymphomas are infrequent (about 3.6% among all extranodal lymphomas) [2]. Primary pulmonary lymphoma (PPL) is defined as a clonal lymphoid proliferation affecting one or both lungs (parenchyma and/or bronchi) in a patient with no detectable extrapulmonary involvement at diagnosis or during the subsequent 3 months [6,7]. According to the World Health Organization’s classification system, the most common histological subtypes of primary pulmonary lymphoma are low-grade lymphomas (75-88%), and the second most frequent histological type of lymphoma to involve the lung is diffuse large B-cell lymphoma (DLBCL), which represents only about 5-20% of primary pulmonary lymphomas [8]. The high-grade B-cell PPLs usually spread rapidly into mediastinal and extra-thoracic locations. The case of DLBCL we reported here presented as a huge lung tumor mimicking a primary lung cancer involving the superior vena cava.

Primary pulmonary lymphoma is most commonly seen in between the 5th and 7th decades of life, with a mean age of 50 years and a male-to-female ratio of 1.07:1 [9]. The present case was a young (25-year-old) man with nonspecific symptoms of irritating dry cough and right chest pain. As reported previously, PPLs can present with nonspecific symptoms [10]. Since PPLs can present with different radiological manifestations, an accurate diagnosis is often not easy. In our case, the images were suggestive of locally advanced lung cancer, but the final diagnosis turned out to be a primary pulmonary lymphoma. This further emphasizes the importance of histological diagnosis of lung lesions, although this tumor was even misdiagnosed as lung cancer before operation based on pathological findings from CT-guided fine-needle aspiration biopsy. In the literature, preoperative CT-guided fine-needle aspiration biopsy allows diagnosis of PPL in only 25% of patients [11]. Thus, in order to avoid the mistake of the misdiagnosis of this curable disease, it is important to keep in mind that any radiological abnormality of the lung parenchyma may be a lymphoproliferative disorder, which sometimes has a good prognosis and has a very different management plan from epithelial neoplasia of the lung, especially when the lung is primarily affected [12].

High-grade pulmonary B-cell lymphoma is far rare and usually occurs in individuals with an underlying disorder (e.g., immunodeficiency). The prognosis is poor compared with low-grade lymphoma, and therapeutic options depend on the underlying disorder. Because of its rarity, relatively little is known about the biological characteristics of DLBCL. Treatment of these rare tumors is poorly standardized. Several treatment options are available, including tumor resection, surgery with adjuvant chemotherapy, or chemotherapy alone. The optimal treatment remains controversial, but the prognosis can be relatively excellent [13]. One large series of 70 patients reported a 94% survival at 5 years for low-grade primary pulmonary lymphoma, and a median survival of 3 years for high-grade disease [10]. Another series with 48 patients reported a 68% 5-year survival for primary pulmonary MALT lymphoma and a 65% 5-year survival for non-MALT lymphoma [3].

We believe surgery should be the treatment of choice if a complete resection can be achieved. The potential surgical candidate could be any patient with locally resectable tumor up to stage IIE. Lymph node involvement does not appear to be a contraindication to surgery [14]. To our knowledge, few patients with pulmonary DLBCL involving the superior vena cava have received radical surgical treatment, so this is an extremely uncommon case. Although some authors have recommended a pneumonectomy for multiple lesions of low-grade PPL involving one lung, in our view this option may be too aggressive because of the indolent course of the disease. One patient reported by Frederic et al. [15] is still alive after a 36-month follow-up.

Treatment after surgical resection is often based on a combination chemotherapy regimen. Diffuse large B cell lymphoma is frequently treated by CHOP (cyclophosphamide, Adriamycin, vincristine and prednisone), and Rituximab may improve the response to CHOP treatment, as has been shown in systemic diffuse large B-cell lymphoma. Although survival is worse for patients with high-grade PPL than for those with low-grade PPL, published reports show that more than half of patients with high-grade PPL can achieve survivals of 8 to 10 years [16].

## Conclusion

In conclusion, surgery should be the treatment of choice in cases of localized PPL when complete resection can be achieved.

## Consent

Written informed consent was obtained from the patient for publication of this Case report and any accompanying images.

## Competing interests

The authors declare that they have no competing interests.

## Authors’ contribution

SW performed research, analyzed data, and wrote the paper; XL, HLZ, XMQ, and GC collected the data and followed up the patient; JC performed operation and research, analyzed data, and wrote the paper; QHZ performed operation and revised the manuscript. All authors read and approved the final manuscript.
